# Living near the port area is associated with physical inactivity and sedentary behavior: Morar perto da área portuária está associado à inatividade física e comportamento sedentário

**DOI:** 10.1590/1516-3180.2016.0151121016

**Published:** 2017-01-05

**Authors:** Evandro Fornias Sperandio, Rodolfo Leite Arantes, Tsai Ping Chao, Marcello Romiti, Antônio Ricardo de Toledo Gagliardi, Victor Zuniga Dourado

**Affiliations:** I PT, PhD. Associate Researcher, Department of Human Movement Sciences, Universidade Federal de São Paulo (Unifesp), Santos (SP), Brazil.; II MD, PhD. Researcher, Department of Cardiovascular Medicine, Angiocorpore Institute of Cardiovascular Medicine, Santos (SP), Brazil.; III PT. Specialization Student, Instituto do Coração (InCor), São Paulo (SP), Brazil.; IV PT, PhD. Associate Professor, Department of Human Movement Sciences, Universidade Federal de São Paulo (Unifesp), Santos (SP), Brazil. Visiting Scholar, Bernard Lown Scholars in Cardiovascular Health Program, Harvard T.H. Chan School of Public Health, Boston, United States.

**Keywords:** Environmental health, Motor activity, Sedentary lifestyle, Social class, Risk factors

## Abstract

**CONTEXT AND OBJECTIVE:**

The impact of the port of Santos, Brazil, on the population’s health is unknown. We aimed to evaluate the association between living near the port area and physical inactivity and sedentary behavior.

**DESIGN AND SETTING:**

Cross-sectional study developed at a university laboratory and a diagnostic clinic.

**METHODS:**

553 healthy adults were selected and their level of physical activity in daily life was assessed using accelerometers. Multiple linear and logistic regressions were performed using physical inactivity and sedentary behavior as the outcomes and living near the port area as the main risk factor, with adjustments for the main confounders.

**RESULTS:**

Among all the participants, 15% were resident near the port area. They took 699 steps/day and presented, weekly, 2.4% more sedentary physical activity, 2.0% less time in standing position and 0.9% more time lying down than residents of other regions. Additionally, living near the port area increased the risk of physical inactivity by 2.50 times and the risk of higher amounts of sedentary behavior (≥ 10 hours/day) by 1.32 times.

**CONCLUSION:**

Living near the port of Santos is associated with physical inactivity and higher sedentary behavior among adults, regardless of confounders. The reasons for this association should be investigated in longitudinal studies.

## RESUMO

**CONTEXTO E OBJETIVOS:**

O impacto do porto de Santos, no Brasil, sobre a saúde da população é desconhecido. Nosso objetivo foi avaliar a associação entre viver nas proximidades da área portuária e a inatividade física e comportamento sedentário.

**TIPO DE ESTUDO E LOCAL:**

Estudo transversal desenvolvido em laboratório universitário e em uma clínica de diagnósticos.

**MÉTODOS:**

Foram selecionados 553 adultos saudáveis e seu nível de atividade física na vida diária foi avaliado usando acelerômetros. Foi realizada regressão linear múltipla e logística usando a inatividade física e o comportamento sedentário como desfechos e morar perto da área portuária como o fator de risco principal, ajustando para os principais confundidores.

**RESULTADOS:**

Entre todos os participantes, 15% residiam na área portuária. Estes deram 699 passos/dia a menos e apresentaram, por semana, 2,4% da atividade física mais sedentária, 2,0% menos tempo em pé e passaram 0,9% mais tempo deitados do que os residentes das demais regiões. Além disso, morar nas proximidades da área portuária aumentou o risco de inatividade física em 2,5 vezes, assim como o risco de maior comportamento sedentário (≥ 10 horas/dia) em 1,32 vezes.

**CONCLUSÃO:**

Morar perto do porto de Santos tem associação com a inatividade física, assim como o aumento do comportamento sedentário em adultos, independentemente de fatores de confusão. As razões para tal associação devem ser investigadas em estudos longitudinais.

## INTRODUCTION

Historically, ports are considered to be engines of economic development for the cities and regions where they are located. The port of Santos in Brazil is one of the most important ports in Latin America due to its size and export capacity.^1^ This is the main gateway for incoming and outgoing products in this country. Despite boosting the economy, it is known that ports cause a negative impact on the health of residents of the surrounding areas.^2^ Living near the port area is associated with low socioeconomic status,^3^ and the pollution of the port increases the risk of developing respiratory^4^ and cardiovascular disease.^5^

According to the global recommendations on physical activity for health, “adults aged 18-64 should do at least 150 minutes of moderate-intensity aerobic physical activity throughout the week or do at least 75 minutes of vigorous-intensity aerobic physical activity throughout the week or an equivalent combination of moderate and vigorous-intensity activity.”^6^ Thus, physical inactivity is characterized as failure to reach the recommendations mentioned above.^7^ Sedentary behavior, in turn, can be defined as “any wakeful behavior characterized by energy expenditure of 1.5 or fewer metabolic equivalent tasks (METs) while in a sitting or reclining posture”.^8^ It is well known that physical inactivity is related to health impairments, but sedentary behavior has recently emerged as a new independent risk factor for chronic diseases as well as for mortality, regardless of moderate-to-vigorous physical activity.^9^^,^^10^^,^^11^^,^^12^^,^^13^^,^^14^ Examples of sedentary behavior include watching television, sitting, playing video games and using computers.^15^ Current studies have been investigating associations of physical activity and sedentary behaviors separately or combined.

Our previous results showed that the proportion of physically inactive subjects in a sample in the city of Santos was between 14% and 20% and that there was an association between physical inactivity and restrictive lung patterns detected by spirometry.^16^^,^^17^ The level of physical activity in daily life is influenced by the physical environment in which subjects live, with their social and individual correlates,^18^ but may also be related to chronic exposure to air pollutants. The vicinity of the port area in Santos seems to be a violent area with few or no safe public spaces where people can perform physical activities. Moreover, it is a highly polluted area, where the annual average levels of particulate matter grossly exceed what is recommended by the World Health Organization.^19^

Information about the impact of the port of Santos on the population’s health is scarce, especially in relation to the level of physical activity within daily life and sedentary behavior directly evaluated by means of triaxial accelerometers. Our hypothesis was that living in neighborhoods close to the port of Santos would be associated with higher prevalence of physical inactivity and increased levels of sedentary behavior, regardless of the main confounders.

## OBJECTIVE

We aimed to evaluate the association between living near the port of Santos and physical inactivity and sedentary behaviors among adults.

## METHODS

### Participants and design

Five hundred and fifty-three adults (≥ 20 years of age) were selected from the Epidemiology and Human Movement Study, i.e. the EPIMOV (Estudo Epidemiológico sobre o Movimento Humano) study. Briefly, the EPIMOV study is an ongoing cohort study with the primary objective of investigating the longitudinal association of sedentary behaviors and physical inactivity with occurrences of hypokinetic diseases, especially cardiorespiratory and musculoskeletal diseases. The present study is a cross-sectional study from the first year of the EPIMOV study. The volunteers who participated in it were recruited through publicity in social networks, folders displayed in the universities of the region, local magazines and newspapers.

We divided the participants into two groups: people residing near the port area and people residing in other surrounding neighborhoods within the metropolitan area of Santos. We used the map of the city to select residents of neighborhoods that are adjacent to the port area. We defined the participants’ socioeconomic level according to the mean income of each neighborhood based on official documents held by the city of Santos, which include a map of the city according to the average income of heads of households. The participants were divided into three monthly income levels (i.e. low: R$ 622-1866; moderate: R$ 1866-3732; and high: R$ 3732-6220).

In the early clinical evaluation, personal and demographic data were collected. In addition, the participants answered the physical activity readiness questionnaire^20^ in order to evaluate some possible risks relating to performing physical exercises such as cardiopulmonary exercise testing. They also answered questions about any history of respiratory illness, based on the American Thoracic Society questionnaire,^21^ to investigate exposure to pollutants, history of asthma and smoking status; and cardiovascular disease risk stratification was performed as specified by the American College of Sports Medicine.^22^

We excluded participants with a self-reported diagnosis of heart disease, lung disease or musculoskeletal disorders. We made objective measurements to evaluate physical activity in daily life through triaxial accelerometry and lung function through spirometry; and conducted cardiopulmonary exercise testing using a ramp protocol on a treadmill. We also investigated the presence of self-reported major risk factors for cardiovascular disease, including age (≥ 45 years for males and ≥ 55 years for females), systemic arterial hypertension, diabetes/hyperglycemia, dyslipidemia/hypercholesterolemia, current cigarette smoking and family history of premature coronary heart disease. A family history of premature coronary heart disease was defined as myocardial infarction or sudden death of father or other male first-degree relative before 55 years of age, or of mother or other female first-degree relative before 65 years of age. Education level was reported as illiterate or completed primary, secondary or tertiary education.

Smoking was also investigated through self-reporting. The subjects were considered to be smokers if they reported current tobacco use and had smoked 100 or more cigarettes during their lifetime.^23^

The participants were informed about the possible risks and discomforts of this study and signed a consent form. The local Ethics Committee for Human Research approved this study (protocol: 186.796).

### Anthropometric measurements

Body weight and height were measured, and the body mass index was calculated in accordance with standardized methods.^24^

### Spirometry

Spirometry was performed using a handheld spirometer (Quark PFT/CPET, Cosmed, Pavona di Albano, Italy) in accordance with the criteria established by the American Thoracic Society.^25^ The forced expiratory volume in the first second (FEV_1_), forced vital capacity (FVC) and FEV_1_/FVC ratio were quantified. The predicted values were calculated using national reference equations.^26^

### Cardiorespiratory fitness

The maximum/symptom-limited exercise capacity was assessed during cardiopulmonary exercise testing on a treadmill (ATL, Inbrasport, Curitiba, Brazil), following a ramp protocol. After 3 minutes at rest, the speed and inclination were automatically incremented according to the estimated maximal oxygen consumption (V’O_2_max), with the aim of completing the test in about 10 minutes.^27^^,^^28^ Cardiovascular, ventilatory and metabolic variables were analyzed breath by breath, using a gas analyzer (Quark PFT, Cosmed, Pavona di Albano, Italy). Oxygen uptake (V’O_2_), carbon dioxide production (V’CO_2_), minute ventilation (V’E), and heart rate were monitored throughout the test. The data were filtered every 15 seconds for further analysis. Peak V’O_2_ was defined as the arithmetic average of the last 15 seconds at the end of the incremental phase of the cardiopulmonary exercise testing.

### Accelerometer-based sedentary behavior and physical activity in daily life

Sedentary behavior and physical activity in daily life were evaluated using a previously validated triaxial accelerometer (ActiGraph GT3X+, MTI, Pensacola, FL, USA).^29^^,^^30^^,^^31^ The equipment consisted of a small, lightweight box (4.6 cm x 3.3 cm x 1.5 cm) that was attached to the waist above the dominant hip, by means of a band (total weight = 19 g). It had the capacity to measure human movement along the vertical, sagittal and mediolateral axes. The participants were subjected to seven consecutive days of evaluation during their wakeful hours. To be considered valid, data collection days needed to have at least 10 hours of continuous monitoring, starting when the subject woke up, together with absence of excessive counts (> 20,000). We instructed the participants to remove the accelerometer at bedtime and during showers and aquatic activities. 

Periods with fewer than 60 counts per minutes (cpm) on the accelerometer were interpreted as periods when the accelerometer was not worn, with a tolerance of 2 minutes for periods with some movement, i.e. less than 50 cpm. The thresholds for the intensity of the physical activity were as follows:^32^ 1. very light (100-759 cpm); 2. light (760-1951 cpm); and 3. moderate-to-vigorous (> 1951 cpm). The minimum quantity and intensity levels for physical activity to be considered as such was 150 minutes of moderate-to-vigorous physical activity per week.^33^^,^^34^ Individuals who did not reach this level of physical activity were considered to be physically inactive.

The total amount of sedentary behavior was determined based on the number of minutes with counts less than 100. On the other hand, active time was considered to be time spent on activities with ≥ 100 cpm. By means of the inclinometer located inside the accelerometer, the time spent in each body position was measured (i.e. reclining during wakeful hours, sitting or standing). The measurements were calculated as minutes/week and as percentages of the total time. Sedentary behavior was also assessed as a categorical variable in accordance with the threshold recently described.^13^^,^^14^ Participants who performed ≥ 10 hours/day of sedentary activities were classified in a group with a high amount of sedentary behavior, whereas the group with a low amount was defined as < 10 hours/day of such activities. Only data from the participants who used the accelerometer for at least four valid days were analyzed.

### Statistical analysis

The sample size was calculated in accordance with the prevalence of physical inactivity of around 20% that was observed in previous findings from the EPIMOV study in the metropolitan area of the city of Santos.^16^ Through taking a 99% confidence interval, it was found that at least 423 participants needed to be enrolled in the present study. We performed the sample size calculation using the free tools available on the website www.openepi.com.

Our first statistical analysis was a descriptive analysis of the data. We then evaluated whether being a resident in the port area was associated with physical inactivity in daily life and sedentary behavior, by means of multiple linear regression, regardless of socioeconomic and educational level. We developed two multiple logistic regression models in which physical inactivity and sedentary behavior were taken to be the outcomes and living near the port area was the main exposure. Adjusted odds ratios and 95% confidence intervals were calculated. Both multiple logistic regressions were adjusted according to the following: age; sex; race (i.e. categorized as black, white, mixed, Amerindian or East Asian); education level (i.e. classified as tertiary educational attainment or not); self-reported cardiovascular disease risk factors (i.e. hypertension, diabetes, dyslipidemia, smoking, obesity or physical inactivity); cardiorespiratory fitness (peak V’O_2_ [ml/min/kg])]; and lung function (FEV_1_ [liters]). Obesity was categorized as yes or no (body mass index ≥ 30 or < 30 kg/m^2^, respectively). The probability of alpha error was set at 5%.

## RESULTS

Fifteen percent (n = 83) of our participants were residents in the port area. These were significantly younger and had higher socioeconomic status (Table 1). However, the univariate analysis showed that sex, race, anthropometry, lung function, exercise capacity, smoking status, physical inactivity and risk of cardiovascular disease variables were not statistically different between residents and non-residents in the vicinity of the port. The prevalences of diabetes mellitus, hypertension and dyslipidemia in this study were similar to those found in population-based studies in Brazil.

**Table 1:**
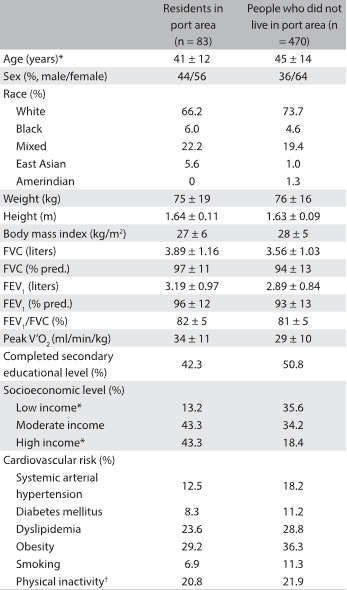
General characteristics of the sample

Data presented as mean ± standard deviation or as count and percentage. *P < 0.05: residents of the port area versus residents of other neighborhoods; †Assessed using triaxial accelerometers. FVC = forced vital capacity; FEV1 = forced expiratory volume in the first second; V’O2 = oxygen uptake.

The results from the linear multiple regression analysis showed that there was an association between living near the port area and increased sedentary behavior, as evaluated using triaxial accelerometers. Other variables such as socioeconomic status, education level and smoking were also significant determinants of higher amounts of sedentary behavior (Table 2). Living in the port area increased the risk of physical inactivity more than twofold, independently of any other confounder. Age and smoking also increased the risk of physical inactivity, after adjusting the logistic regression model according to age, gender, education level, socioeconomic status, risk factors for cardiovascular disease, cardiorespiratory fitness, lung function and smoking. On the other hand, cardiorespiratory fitness reduced the risk of physical inactivity (Table 3).

**Table 2:**
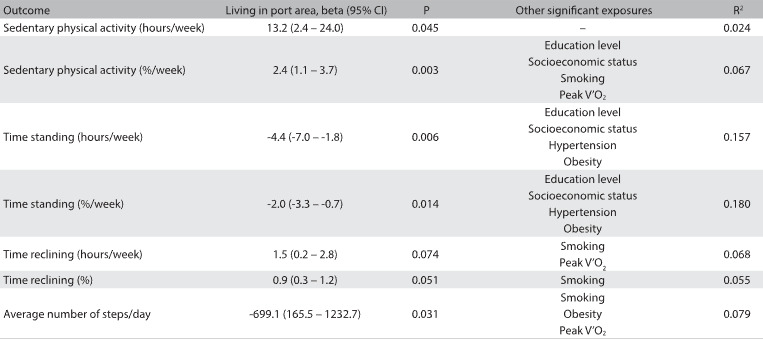
Results from linear multiple regression analysis on the association between sedentary behavior evaluated using accelerometers and living in the port area

CI = confidence interval. Models adjusted for age, gender, education level, socioeconomic status, hypertension, diabetes mellitus, dyslipidemia, obesity, cardiorespiratory fitness, lung function and smoking.

**Table 3:**
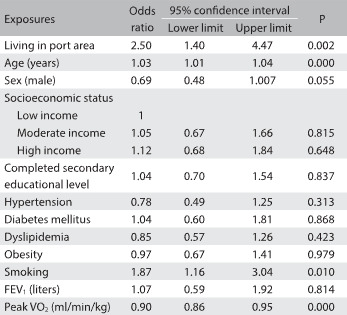
Results from the logistic regression analysis between physical inactivity assessed using accelerometers and factors associated to it (exposures)

Models adjusted for age, gender, education level, socioeconomic status, hypertension, diabetes mellitus, dyslipidemia, obesity, smoking, lung function and cardiorespiratory fitness. FEV1 = forced expiratory volume in the first second; V’O2 = oxygen uptake.

Regarding sedentary behavior, 51.7% of our participants performed ≥ 10 h/day of sedentary activities. Living near the port increased the risk of high amounts of sedentary behavior by 32%. In this multiple logistic regression model, age, gender, socioeconomic status, education level and smoking were also selected as determinants of high amounts of sedentary behavior. There was a positive association between higher socioeconomic status and higher amounts of sedentary behavior (Table 4).

**Table 4:**
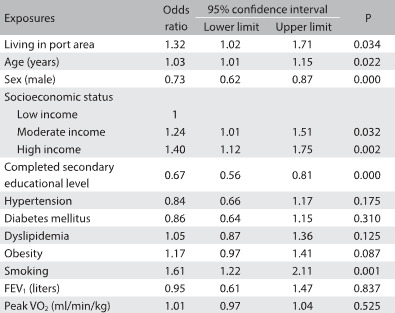
Results from the logistic regression analysis between sedentary behavior assessed by accelerometers and factors associated to it (exposures)

Sedentary behavior: categorized as high (≥ 10 hours/day) or low (< 10 hours/day). Models adjusted for age, gender, education level, socioeconomic status, hypertension, diabetes mellitus, dyslipidemia, obesity, smoking, cardiorespiratory fitness and lung function. FEV1 = forced expiratory volume in the first second; V’O2 = oxygen uptake.

Through multiple regression analysis, the residents of the port area showed higher amounts of sedentary behavior, i.e. less time standing and more time reclining, and also a lower number of steps/day, in comparison with people who did not live in the port area (Table 5).

**Table 5:**
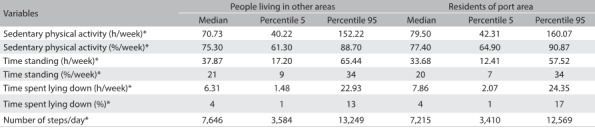
Comparison between residents of the port area and people living in other areas regarding sedentary behaviors and the number of steps/day

*P < 0.05: residents of the port area versus residents of other neighborhoods.

## DISCUSSION

This study investigated the association between living near the largest port in Latin America and physical inactivity and sedentary behavior among adults. The associations found indicated that living near the port of Santos increased the risk of physical inactivity and sedentary behavior among adults, regardless of socioeconomic status, education level, cardiovascular risk, lung function or cardiorespiratory fitness.

Unlike what we expected, the residents of the port area were younger and had higher socioeconomic status than people who did not live in the port area. These results contrast with previously published data. Grobar^3^ observed that the unemployment and poverty rates are significantly higher in port districts. This disparity is possibly due to a peculiarity of the city of Santos. The neighborhood of Ponta da Praia, one of the neighborhoods with the highest average income of the city, is located very close to one of the main terminals of the port. Nevertheless, living near the port region increased the risk of physical inactivity and sedentary behavior, regardless of the higher socioeconomic status of the residents of Ponta da Praia. This finding is interesting because studies have shown that low socioeconomic status groups perform an insufficient amount of physical activity to achieve health benefits.^35^ Our results suggest that living next to a major port could affect lifestyle, even among people with privileged socioeconomic status in relation to Brazilian patterns. Therefore, whether living in the port area in Santos is different from living in another port area elsewhere in the world remains to be clarified.

Although there was no association between socioeconomic status and physical inactivity, we observed a positive association between higher socioeconomic status and higher amounts of sedentary behavior. It has been suggested that the associations between socioeconomic status and sedentary behavior present different directions in high-income countries, compared with low and middle-income countries, and that this varies according to the domain of sedentary behavior. Overall, the association between socioeconomic level and sedentary behavior is inverse.^36^ However, Mielke et al.^36^ observed that this relationship varies according to the income level of the country. In high-income countries, socioeconomic status presented an inverse association with sedentary behavior (effect size: 0.67; 95% CI: 0.62-0.73), whereas a positive relationship was observed in low to middle-income countries (effect size: 1.18; 95% CI: 1.04-1.34). Unlike in high-income countries, in which all indicators of socioeconomic level were negatively associated with sedentary behavior, only resources showed a significant positive association in low to middle-income countries. Despite the significant relationship mentioned above, living in the port area remained a significant determinant of higher amounts of sedentary behavior.

Residents near port areas are exposed to increased levels of air pollution due to emissions of particulate matter derived from the exhaust fumes of trucks and ships, and as a result of mechanical processes of milling operations and the ensuing street dust suspensions. Very recent studies have reported on the influence of air pollution on decreased physical activity.^37^^,^^38^^,^^39^ In one of these studies, particulate matter and O_3_ levels were correlated with reduction in physical activity in daily life and the number of steps/day, among patients with chronic obstructive pulmonary disease (COPD).^38^ Although air pollution was not assessed in our study, we believe that this in the port of Santos may partly explain the higher proportion of physically inactive people and larger amount of sedentary behavior among residents of the port area. In fact, a recent large study conducted in Brazil showed that the particulate matter monitoring in the city of Santos is poor and started only in 2011. Moreover, Santos only has two air-monitoring stations and is classified as having the sixth highest concentration of particulate matter in the state of São Paulo, Brazil. The average level of particulate matter in the metropolitan area of the city of Santos was 37.23 µg/m^3^ (annual mean) in 2011, which was significantly above the levels recommended by the World Health Organization. Despite the lack of assessment of particulate air pollution in the present study, it would be rational to suppose that environmental exposure to particulate matter may play a major role in the results presented here.^19^

Our results also showed that smoking was associated with physical inactivity and with greater amounts of sedentary behavior, independently. Previous results from the EPIMOV study^40^ reinforce the findings of the present study. We compared two groups of physically active individuals, one formed by smokers and the other by nonsmokers. Although they performed the same amount of moderate-to-vigorous physical activity, as assessed directly using triaxial accelerometers, and were matched regarding major confounders, the smokers performed higher amounts of sedentary physical activity and spent more time sitting and lying down per week. Like in the present study, other recent studies have reported an association between smoking and physical inactivity.^41^^,^^42^

As we expected, cardiorespiratory fitness was inversely associated with physical inactivity and living near the port did not alter the risk of physical inactivity. Ecological models for physical activity and sedentary behavior identified influences from several attributes, including individual components, the social environment, the physical environment and public policy. Some of the main barriers preventing physical activity are lack of motivation, awareness and time, and lack of structure for physical activity.^43^ People may have the necessary knowledge, skills, attitudes and motivation to be physically active, but if they do not have access to the necessary opportunities, they may be restricted or prohibited from being active. Building or enhancing facilities for physical activity can require a large amount of time and resources. Public health policies and intervention programs designed with a focus on increasing the level of physical activity and decreasing sedentary behavior are probably necessary for this region of Santos. Regarding the determinants of physical inactivity and sedentary behavior, cohort studies are needed to investigate the causes of the associations of physical inactivity and greater amounts of sedentary behavior with living near the port area of Santos.

This study has limitations that need to be described. The cross-sectional design did not allow us to establish any relationship between cause and effect. However, our objective was to evaluate the association between living near the port area of Santos and physical inactivity and sedentary behavior. We found that these associations were consistent. Our findings may guide new research questions towards identifying other determinants of physical inactivity and sedentary behavior relating to major ports.

## CONCLUSIONS

Living near the largest port in Latin America, located in the city of Santos, Brazil, is associated with physical inactivity and sedentary behavior among adults, regardless of socioeconomic status, education level, cardiovascular risk, lung function or cardiorespiratory fitness. Whether this association is related to environmental exposure and/or to lack of equipment for physical activity in this region should be investigated in cohort studies.
